# Cellular and humoral immunity of virus-induced asthma

**DOI:** 10.3389/fmicb.2013.00252

**Published:** 2013-08-27

**Authors:** Yoshimichi Okayama

**Affiliations:** Allergy and Immunology Group, Research Institute of Medical Science, Nihon University School of MedicineTokyo, Japan

**Keywords:** respiratory syncytial virus, human rhinovirus, virus-induced asthma, cellular immunity, humoral immunity

## Abstract

Asthma inception is associated with respiratory viral infection, especially infection with respiratory syncytial virus (RSV) and/or human rhinovirus (HRV), in the vast majority of cases. However, the reason why RSV and HRV induce the majority of bronchiolitis cases during early childhood and why only a small percentage of children with RSV- and HRV-induced bronchiolitis later develop asthma remains unclear. A genetic association study has revealed the important interaction between viral illness and genetic variants in patients with asthma. Severe RSV- and HRV-induced bronchiolitis may be associated with a deficiency in the innate immune response to RSV and HRV. RSV and HRV infections in infants with deficient innate immune response and the dysfunction of regulatory T cells are considered to be a risk factor for the development of asthma. Sensitization to aeroallergens, beginning in the first year of life, consistently predisposes children to HRV-induced wheezing illnesses, but the converse is not true. Some evidence of virus specificity exists, in that allergic sensitization specifically increased the risk of wheezing in individuals infected with HRV, but not RSV. Administration of Palivizumab, a humanized monoclonal antibody that targets the A antigenic site of the Fusion-protein of RSV, decreases the risk of hospitalization in high-risk infants and the risk of recurrent of wheezing. However, palivizumab did not have any effect on subsequent recurrent wheezing in children with a family history of atopy. These findings suggest that infection with RSV and infection with HRV might predispose individuals to recurrent wheezing through an atopy-independent and an atopy-dependent mechanism, respectively. Respiratory virus-induced wheezing illnesses may encompass multiple sub-phenotypes that relate to asthma in different ways.

## INTRODUCTION

Infection with respiratory syncytial virus (RSV) and/or human rhinovirus (HRV) is the most important cause of lower respiratory tract disease in infants and young children (Heilman, 1990) and is the major cause of bronchiolitis in infants (Henderson et al., 1979; Rakes et al., 1999). RSV- and/or HRV-induced bronchiolitis during early childhood is strongly linked to the subsequent development of allergies and asthma (Sigurs et al., 2000; Schauer et al., 2002; Lemanske et al., 2005; Kusel et al., 2007; Jackson et al., 2008, 2013; Sly et al., 2008; Stern et al., 2008). However, the reasons why RSV and HRV induce the majority of bronchiolitis cases during early childhood and why only a small percentage of children with RSV- and/or HRV-induced bronchiolitis later develop atopy or asthma remain unclear. Accumulating evidence suggests that both genetic and environmental factors determine the type of immune response to infection with RSV and/or HRV and that the type of immune response may, in turn, affect the development of asthma (Martinez, 2003; Caliskan et al., 2013). This chapter focuses on the immunological mechanisms that may explain why asthma inception is associated with RSV- and HRV-infection.

## INTERACTION BETWEEN VIRAL ILLNESS AND GENETIC VARIANTS IN PATIENTS WITH ASTHMA

Asthma inception and exacerbation are associated with respiratory viral infection in the vast majority of cases (Kusel et al., 2007; Jackson et al., 2008; Sly et al., 2008; Stern et al., 2008). Severe bronchitis following infection with RSV can occur in a small subset of infected infants (Sly et al., 2008). Viral infections of the lower respiratory tract are likely to result in wheezing if they induce inflammation and edema of the airway epithelium, decreasing the airway diameter (Sly et al., 2008). The frequency of lower respiratory viral infection accompanied by wheezing influences the risk of asthma inception (Sly et al., 2008). A genetic association study involving 470 children hospitalized for RSV-induced bronchiolitis, their parents, and 1008 randomly selected population controls showed that single-nucleotide polymorphisms (SNPs) of *VDR* (vitamin D receptor), *JUN*, *IFNA5*, *NOS2A*, and *FCER1A* were significantly associated with severe RSV-induced bronchiolitis at both the allele and genotype levels (Janssen et al., 2007). Genetic polymorphisms of *SFTPA* (surfactant protein A), *SFTPD* (surfactant protein D), *TLR* (toll like receptor) 4, *TNF* (tumor necrosis factor), IL4 (interleukin 4), *IL9, IL10, IL8, IL13, IL4RA,* and *CCL5* have been reportedly associated with a susceptibility to RSV-induced bronchiolitis (Huckabee and Peebles, 2009). Very recently, a significant interaction between the 17q21 genotype and human RSV-induced wheezing illness was demonstrated in two birth cohorts of children: the childhood origins of asthma (COAST), and the Copenhagen Prospective Study on Asthma in Childhood (COPSAC; Caliskan et al., 2013). The effects of 17q21 variants on an increased susceptibility to asthma are restricted to patients with a history of HRV-induced wheezing illness during early life (Caliskan et al., 2013). These studies emphasize the important interaction between virus-induced illness and genetic variants in patients with asthma.

## CELLULAR IMMUNITY OF VIRUS-INDUCED ASTHMA

(1) Is RSV- and/or HRV-induced severe bronchiolitis in children associated with a T helper type 2 (Th2)-predominant immune response?

RSV-induced severe bronchiolitis in children is associated with a Th2-predominant immune response (Renzi et al., 1997; Roman et al., 1997; Bendelja et al., 2000; Pala et al., 2002; van der Sande et al., 2002; Joshi et al., 2003; Legge and Braciale, 2003) or a decreased Th1 immune response (Joshi et al., 2003; Legge and Braciale, 2003). The concentration of Th2 cytokines was higher than that of interferon (IFN)-γ in nasopharyngeal secretions (NSP; Bermejo-Martin et al., 2007), particularly in infants less than three months old (Kristjansson et al., 2005). On the other hand, undetectable or very low Th2 cytokine concentrations have also been reported (Bont et al., 2001; Garofalo et al., 2001; Bennett et al., 2007; Garcia et al., 2012).

The majority of virus-infected mouse studies have been performed using RSV because the major group of HRV (88% of known HRV serotypes) uses human intercellular adhesion molecule-1 (ICAM-1) as a cellular receptor and cannot bind to mouse ICAM-1. Regarding HRV-induced asthma mouse models, three novel mouse models of HRV infection have been recently developed: infection with a minor-group HRV (the receptor is the low-density lipoprotein receptor family) in BALB/c mice, infection with a major-group HRV in transgenic BALB/c expressing a mouse-human ICAM-1 chimera, and HRV-induced exacerbation of allergic airway inflammation (Bartlett et al., 2008). These models are likely to be useful for the future development of therapies for asthma exacerbation.

In the majority of RSV-infected mouse studies, the induction of Th2 cytokines (including IL-4 and IL-5) is not observed in bronchial alveolar lavage (BAL) fluid or the lung tissues of RSV-infected mice (Peebles et al., 2001; Chavez-Bueno et al., 2005; Lee et al., 2008). A comparison of mouse strains showed that BALB/c and DBA/2 mice had a significantly higher airway hyper-reactivity over almost the entire time course up to 20 days after RSV exposure, compared with C57BL/6 mice (Tekkanat et al., 2001). However, even the BALB/c mice required a very high intranasal or intratracheal inoculum (10^5^ or 10^6^ PFUs) to elicit airway hyper-responsiveness (AHR; Tekkanat et al., 2001; Wang et al., 2004). Therefore, both the mouse strains and the amounts of virus that were used should be taken into consideration when comparing different mouse experiments.

(2) Is severe RSV- and/or HRV-induced bronchiolitis associated with deficient IFN production?

The concentrations of IFN-γ in samples of blood mononuclear cells or nasopharyngeal aspirates from RSV- and/or HRV-infected infants were inversely correlated with disease severity (Aberle et al., 1999; Bont et al., 1999, 2001; Renzi et al., 1999; Legg et al., 2003; Bennett et al., 2007; Garcia et al., 2012). These clinical studies suggest that IFN-γ plays an important role in determining the severity of RSV- and/or HRV-induced bronchiolitis.

STAT1-knockout (STAT1^-^^/^^-^) BALB/c mice, which are incapable of responding to type II IFN (IFN-β) or to type I IFN (IFN-α and β ), showed markedly increased levels of inflammation, compared with wild-type (WT) mice, after infection with RSV, despite similar virus titers in the lung and similar rates of viral clearance (Durbin et al., 2002). STAT1^-^^/^^-^ mice, but not WT or IFN-γ^-^^/^^-^-infected mice, exhibited eosinophilic and neutrophilic pulmonary infiltrates. Although IFN-γ had been induced in infected lung tissues from both STAT1^-^^/^^-^ and WT mice, preferential IL-4, IL-5, and IL-13 induction was only seen in the STAT1^-^^/^^-^ mice. These findings suggest that both type I and type II IFNs play important roles in the Th1 antigen-specific immune response to RSV infection (Durbin et al., 2002).

(3) Is infection with RSV and/or HRV during infancy a risk factor for the development of asthma and possible allergic sensitization?

Among the viral wheezing illnesses that occur in outpatients during infancy and early childhood, those caused by HRV infections are the most significant predictors of the subsequent development of asthma at an age of 6 years, with an odds ratio (OR) of 9.8 for a high-risk birth cohort (Jackson et al., 2008). From birth to 3 years of age, wheezing accompanied by RSV infection alone was associated with an increased asthma risk at an age of 6 years, with an OR of 2.6 (Jackson et al., 2008). Two important observations must be considered. First, most severe RSV infections occur between the 8th and 24th postnatal week. 80% of infants experience an HRV infection by the age of 1 year. Second, repeated infection is common at all ages, indicating the possibility of important interactions and outcomes in RSV- and/or HRV-re-infected hosts.

Culley et al. (2002) compared the consequences of re-infection with RSV between neonatal mice (during the 1 week of life) and weanling mice (at 3 weeks of age) that had been initially infected with RSV. The primary infection of neonatal BALB/c mice with RSV was associated with a reduced and delayed IFN-γ response (Culley et al., 2002). Upon re-infection with RSV, a severer weight loss with the increased inflammatory cell recruitment of Th2 cells and eosinophils was induced in the neonatal mice, compared with the weanling mice that had been initially infected with RSV (Culley et al., 2002). These results show the crucial importance of age at the time of the first infection in determining the outcome of re-infection and suggest that the environment of the neonatal lung is a major determinant of cytokine production and disease patterns in later life. Neonatal RSV infection is thought to lead to a predisposition to the development of airway eosinophilia and enhanced AHR via an IL-13-dependent mechanism during re-infection, whereas infection at a later age protects against the development of these altered airway responses after re-infection (Dakhama et al., 2005).

Severe RSV bronchiolitis has been associated with deficient IFN-γ production in humans (Bont et al., 2001; Bennett et al., 2007; Garcia et al., 2012), but the role of this cytokine in determining the outcome of re-infection is unknown. To define the role of IFN-γ in the development of RSV-induced AHR and lung histopathology in mice, WT and IFN-γ^-^^/^^-^ mice were infected with RSV at a newborn or weaning stage and were re-infected 5 weeks later. (Lee et al., 2008). Both WT and IFN-γ^-^^/^^-^ mice developed similar levels of AHR and airway inflammation after the primary infection (Lee et al., 2008). After re-infection, the IFN-γ^-^^/^^-^ mice, but not the WT mice, developed AHR, airway eosinophilia, and mucus hyperproduction. The intranasal administration of IFN-γ during the primary infection, but not during the re-infection, prevented the development of these altered airway responses upon re-infection in the IFN-γ^-^^/^^-^ mice. IFN-γ production during primary RSV infection is critical to the development of protection against AHR and airway eosinophilia as well as mucus hyperproduction during subsequent re-infection (Lee et al., 2008).

To define the mechanism underlying the enhanced responsiveness in neonatally infected mice that were re-infected with RSV, the differences between dendritic cells (DCs) from neonatal mice and those from 5-week-old mice were examined. Neonatal lung MHCII-positive (^+^) CD11b^+^ DCs expressed higher baseline levels of OX40 ligand (OX40L) and lower cytoplasmic levels of IL-12 than lung DCs from 5-week-old mice (Han et al., 2012). Following RSV infection, OX40L expression was increased in neonatal DCs. The administration of anti-OX40L neutralizing antibody during primary RSV infection in neonatal mice prevented the subsequent enhancement of AHR and the development of airway eosinophilia and mucus hyperproduction upon re-infection. The basal expression levels of thymic stromal lymphopoietin (TSLP) in the lungs were higher in the neonates than in the 5-week-old mice (Han et al., 2012). RSV infection upregulates TSLP production in epithelial cells (Han et al., 2012). The administration of anti-TSLP neutralizing antibody before neonatal RSV infection reduced the accumulation of lung DCs, decreased OX40L expression on lung DCs, and attenuated the enhancement of the airway responses after re-infection (Han et al., 2012).

Regulatory T (T_reg_) cells have an important role in immune tolerance as early as the embryonic stage (Mold et al., 2008, 2010). T_reg_ cell-mediated protection from asthma is initiated at the neonatal stage. Airborne antigens and ovalbumin (OVA) are efficiently transferred from mother mice to neonatal mice through transforming growth factor (TGF)-β in lactated milk (Polte and Hansen, 2008; Verhasselt et al., 2008). The repeated RSV infection of infant mice impaired T_reg_ cell function, leading to a malfunction in tolerance to OVA contained in lactated milk. As a result, RSV increased allergic airway inflammation in response to OVA sensitization and subsequent challenges, compared with inflammation in uninfected, tolerant control mice (Krishnamoorthy et al., 2012). Virus infection induced GATA-3 expression and Th2 cytokine production in forkhead box P3 (FOXP3)^+^ T_reg_ cells and compromised the suppressive function of pulmonary T_reg_ cells. These findings highlight a mechanism by which viral infection targets a host-protective mechanism during early life and increases susceptibility to allergic disease (Krishnamoorthy et al. 2012).

## HUMORAL IMMUNITY OF VIRUS-INDUCED ASTHMA

Most children experience primary RSV infection when they are infants. The immune system is still immature, and maternally derived antibodies are still present at relatively high levels during this period of life (Brandenburg et al., 1997). The titers of maternally derived neutralizing antibodies are reportedly inversely associated with RSV infection overall (Roca et al., 2002; Ochola et al., 2009) and with the severity of RSV illness (Glezen et al., 1981; Holberg et al., 1991; Piedra et al., 2003; Stensballe et al., 2009). Prophylaxis by Palivizumab, a humanized monoclonal anti-body that targets the A antigenic site of the F-protein of RSV, substantially reduces the risk of hospitalization in high-risk infants (Feltes et al., 2003). However, maternally derived neutralizing antibodies have a relatively weak ability to mount the antibody responses observed in infants (Murphy et al., 1986). Significant increases in serum or nasal wash concentrations of RSV glycoprotein (G protein) or fusion (F) protein-specific IgG and IgA are not seen in all infants after primary RSV infection (Brandenburg et al., 1997; De Alarcon et al., 2001). The response of human neonatal B cells to RSV uses a biased antibody variable gene repertoire that lacks somatic mutations (Weitkamp et al., 2005; Williams et al., 2009). Because of the poor Toll-like receptor stimulation in B cells, antibody affinity maturation is not sufficient to elicit protection against RSV re-infection (Delgado et al., 2009).

Palivizumab prophylaxis decreases the risk of hospitalization in high-risk infants (Feltes et al., 2003) and the risk of recurrent wheezing (Simoes et al., 2007). However, no effect of palivizumab on subsequent recurrent wheezing was seen in children with a family history of atopy or food allergies, compared with untreated infants with an atopic family history (Simoes et al., 2010). This finding suggests that RSV infection predisposes an individual to recurrent wheezing in an atopy-independent manner and that RSV infections of the lower respiratory tract may have differential effects on the development of recurrent wheezing, depending on the genetic predisposition.

Welliver et al. reported the presence of IgE antibodies recognizing whole RSV and purified F and G protein in a high proportion of NPS samples from infants with RSV-induced bronchiolitis, but rarely in samples from infants with other RSV-induced illnesses (Welliver et al., 1981, 1989). Although others have confirmed the presence of RSV-specific IgE serum and NPS from some RSV-infected infants, such findings are generally seen in a small proportion of patients (Bui et al., 1987; Russi et al., 1993; Wilczynski et al., 1994; Rabatic et al., 1997; Aberle et al., 1999) or not at all (De Alarcon et al., 2001).

In a mouse model, primary infection with RSV was capable of leading to the production of RSV-specific IgE, which may contribute to the development of exaggerated Th2-based airway responses upon reinfection in mice initially infected as neonates (Dakhama et al., 2009).

## RELATIONSHIP BETWEEN INNATE IMMUNE RESPONSE TO INFECTION WITH RSV- AND/OR HRV-INDUCED ASTHMA

Wark et al. (2005) examined virus replication and innate responses to RV-16 infection using primary bronchial epithelial cells from asthmatic and healthy control subjects. Viral RNA expression and late virus release into the supernatant was increased by 50- and 7-fold, respectively, in cells from asthmatic individuals, compared with the levels in cells from healthy controls. The profound impairment of virus-induced IFN-β production in cultures of cells from asthmatic individuals was observed. In infected asthmatic cells, exogenous IFN-β induced apoptosis and reduced virus replication, demonstrating a causal link between deficient IFN-β, impaired apoptosis, and increased virus replication. Message et al. (2008) reported that HRV inoculation induced significantly greater lower respiratory symptoms and lung function impairment and increases in AHR and eosinophilic lower airway inflammation in asthmatic individuals, compared with normal volunteers. The virologic and clinical outcomes were strongly related to deficient IFN-γ and IL-10 responses and to augmented IL-4, IL-5, and IL-13 responses. HRV was detected in the lower airway tissue of patients with asthma significantly more often than in non-asthmatic subjects, and its presence was associated with the clinical features of severer disease (Wos et al., 2008).

## CONCLUSION

**FIGURE 1:**
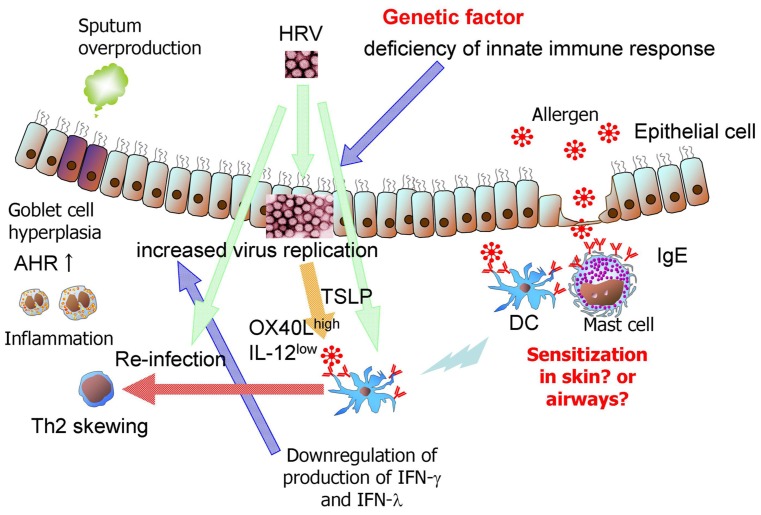
**Possible immunological mechanisms of HRV -induced bronchial asthma in infants.** Sensitization to aeroallergens, beginning in the first year of life, consistently predisposes children to HRV-induced wheezing illnesses. Ongoing allergic inflammation in the airways may directly lead to impairment of the epithelial cell barrier and antiviral response. Following aggregation of Fcε RI, HRV-induced IFN-α and IFN-λ1 production from PBMCs and DCs is significantly reduced compared to that by HRV stimulation alone. Furthermore, impairment of virus-induced IFNs from epithelial cells due to genetic variants increases virus replication. HRV infection upregulates TSLP production in epithelial cells (Kato et al., 2007). Neonatal lung DCs express higher baseline levels of OX40 ligand (OX40L) and lower cytoplasmic levels of IL-12 than lung DCs from adult lung DCs. TSLP upregulates the expression of OX40L on DCs. Airway inflammation and AHR may be enhanced after re-infection

A prospective, repeated characterization of a birth cohort demonstrated that sensitization to aeroallergens, beginning in the first year of life, consistently predisposes children to viral wheezing illnesses and that the converse is not true (Jackson et al., 2013; **Figure 1**). Some evidence suggests virus specificity, in that allergic sensitization specifically increased the risk of wheezing in individuals with HRV infections, but not those with RSV infections. This sequential relationship and the plausible mechanisms by which allergic sensitization can lead to severer HRV-induced lower respiratory illnesses support a causal role for allergic sensitization in this developmental pathway (Jackson et al., 2013). Subrata et al. (2009) suggested that interactions between innate antiviral and allergic inflammatory pathways may lead to severer viral illnesses in atopic children. Ongoing allergic inflammation in the airways may directly lead to impairment of the epithelial cell barrier and the antiviral response (**Figure 1**). Accordingly, respiratory viral infection in atopic children may initiate an atopy-dependent cascade that amplifies and sustains airway inflammation initiated by innate antiviral immunity via harnessing the underlying atopy-associated mechanisms (Subrata et al., 2009). Allergic asthmatic children had higher surface expression of FcεRIα on plasmacytoid (p) DCs and myeloid (m) DCs when compared with that seen in nonallergic, nonasthmatic children. The percentage of FcεRIα ^+^ pDCs and mDCs in allergic asthmatic children was inversely correlated with HRV-induced IFN-α and IFN-λ1, and IFN-α production levels, respectively (**Figure 1**). Following aggregation of Fcε RI, HRV-induced IFN-α and IFN-λ1 production from peripheral blood mononuclear cells (PBMCs) was significantly reduced compared to that by HRV stimulation alone (Durrani et al., 2012). These effects may explain why children with allergic asthma have more frequent and severe HRV-induced wheezing and asthma exacerbations. In contrast, the administration of palivizumab prophylaxis had no effect on subsequent recurrent wheezing in children with a family history of atopy (Simoes et al., 2010). This finding suggests that RSV infection predisposes individuals to recurrent wheezing through an atopy-independent mechanism. Several asthmatic or wheezing phenotypes exist in children (Fitzpatrick et al., 2011). Therefore, respiratory virus-induced wheezing illnesses can encompass multiple sub-phenotypes that relate to asthma in different ways.

## Conflict of Interest Statement

The author declares that the research was conducted in the absence of any commercial or financial relationships that could be construed as a potential conflict of interest.
